# Defective monocyte-derived macrophage phagocytosis is associated with exacerbation frequency in COPD

**DOI:** 10.1186/s12931-021-01718-8

**Published:** 2021-04-20

**Authors:** R. Singh, K. B. R. Belchamber, P. S. Fenwick, K. Chana, G. Donaldson, J. A. Wedzicha, P. J. Barnes, L. E. Donnelly, COPDMAP consortium

**Affiliations:** National Heart and Lung Institute, Imperial College London, London, UK

**Keywords:** COPD, Exacerbation, Macrophage, Lower airway bacterial colonisation

## Abstract

**Background:**

Lower airway bacterial colonisation (LABC) in COPD patients is associated with increased exacerbation frequency and faster lung function decline. Defective macrophage phagocytosis in COPD drives inflammation, but how defective macrophage function contributes to exacerbations is not clear. This study investigated the association between macrophage phagocytosis and exacerbation frequency, LABC and clinical parameters.

**Methods:**

Monocyte-derived macrophages (MDM) were generated from 92 stable COPD patients, and at the onset of exacerbation in 39 patients. Macrophages were exposed to fluorescently labelled *Haemophilus influenzae* or *Streptococcus pneumoniae* for 4 h, then phagocytosis measured by fluorimetry and cytokine release by ELISA. Sputum bacterial colonisation was measured by PCR.

**Results:**

Phagocytosis of *H. influenza*e was negatively correlated with exacerbation frequency (r = 0.440, p < 0.01), and was significantly reduced in frequent vs. infrequent exacerbators (1.9 × 10^3^ RFU vs. 2.5 × 10^3^ RFU, p < 0.01). There was no correlation for *S. pneumoniae*. There was no association between phagocytosis of either bacteria with age, lung function, smoking history or treatment with inhaled corticosteroids, or long-acting bronchodilators. Phagocytosis was not altered during an exacerbation, or in the 2 weeks post-exacerbation. In response to phagocytosis, MDM from exacerbating patients showed increased release of CXCL-8 (p < 0.001) and TNFα (p < 0.01) compared to stable state.

**Conclusion:**

Impaired COPD macrophage phagocytosis of *H. influenza*e, but not *S. pneumoniae* is associated with exacerbation frequency, resulting in pro-inflammatory macrophages that may contribute to disease progression. Targeting these frequent exacerbators with drugs that improve macrophage phagocytosis may prove beneficial.

**Supplementary Information:**

The online version contains supplementary material available at 10.1186/s12931-021-01718-8.

## Background

Lower airway bacterial colonization (LABC) occurs in approximately half of patients with chronic obstructive pulmonary disease (COPD), and is associated with increased inflammation, increased exacerbation frequency and a faster rate of lung function decline [1]. Alveolar macrophages are the main phagocytic cell of the lungs during homeostasis, responsible for the removal of invading pathogens. However, macrophages from patients with COPD display defective phagocytosis of bacteria including *Haemophilus influenzae* and *Streptococcus pneumoniae* [2, 3], apoptotic cells [4] and fungal spores [5] and exhibit a pro-inflammatory secretory phenotype which drives inflammation in the lungs [6]. LABC can persist in the COPD lungs despite a 20-fold increase in the number of alveolar macrophages [7, 8] due to the recruitment of circulating monocytes during inflammation [9, 10], which differentiate into macrophages in the lung and contribute to the macrophage pool. These monocyte-derived macrophages (MDM) also carry the defect in phagocytosis of bacteria [2, 11] and fungi [5] and are utilized as a non-invasive model of alveolar macrophages. The inability of macrophages to clear bacteria may contribute to persistent LABC, however this has not been fully explored.

Exacerbations of COPD are usually associated with viral or bacterial infection, and lead to the acute worsening of symptoms and faster decline in lung function [12]. The contribution of the innate immune system, including macrophages, during exacerbation is not clear due to the difficulty in performing bronchoscopy during these acute episodes. Studies to date have looked at sputum, showing sputum contains higher levels of IL-6 and CXCL-8 at exacerbation [13], while alveolar macrophages taken from stable frequent exacerbator COPD released reduced CXCL-8 and TNFα in response to bacteria compared to infrequent exacerbators, suggesting an association between macrophage function and exacerbation frequency [14]. We have taken this further, and used the MDM model to study macrophage phagocytosis in the stable state, and at the onset of exacerbation, and analyzed whether this is associated with clinical phenotype, LABC status and exacerbation frequency. The influence of these factors may determine patients that would respond to treatments that target macrophage function or exacerbations.

## Methods

### Patient recruitment

Ninety-two COPD patients enrolled in the London COPD cohort between November 2011 and February 2014 were included. Ethical approval was granted from the Hamstead research ethics committee (Ref. 09/H0720/8) and all patients gave written informed consent. Patients were included with a smoking history of > 10 pack years, evidence of persistent airflow obstruction with forced expiratory volume in 1 s (FEV_1_)/forced vital capacity (FVC) < 0.7 in keeping with GOLD grades I-IV [15]. Stable state was defined as without evidence of symptom-defined exacerbations in the preceding 4 weeks and the subsequent 2 weeks post-clinic visit. Clinical demographics can be seen in Table 1.

**Table 1:** Clinical demographics of COPD patient subgroups

	Stable patients n = 92	Repeatability analysis n = 36	Paired exacerbation patients n = 39
Age (years)	71.9 (8.7)	69.3 (8.7)	71.5 (8.0)
FEV_1_ (L)	1.34 (0.52)	1.47 (0.58)	1.27 (0.55)
FEV_1_ (% predicted)	54.6 (17.6)	58.1 (18.4)	52.7 (18.8)
FVC (L)	2.86 (0.87)	2.90 (0.77)	2.69 (0.93)
FEV_1_/FVC (%)	47.6 (13.4)	50.7 (13.1)	48.2 (15.4)
Exacerbation frequency	2.0 [1.0–3.0]	2.0 [1.0–3.0]	2.5 [1.5–3.5]
Smoking pack years	50.0 [28.4–71.5]	45.3 [21.1–62.3]	45.6 [24.5–76.5]
ICS dose (beclomethasone equivalent μg)	1000 [1000–2000]	1500 [1000–2000]	2000 [1000–2000]
N (%) Male sex	60 (65)	24 (67)	23 (59)
N (%) Current smokers	32 (35)	17 (47)	11 (28)
N (%) Chronic bronchitis	72 (78)	31 (86)	35 (90)

Table shows data for stable COPD patients (n = 92), including subsets of patients used for repeatability experiments (n = 36) and patients with a paired stable and exacerbation sample (n = 39). Parametric data displayed as mean (SD), non-parametric data displayed as median (IQR) and categorical data displayed as N (%)

An exacerbation was defined using validated symptomatic criteria [14]. Exacerbation onset was defined as the first day on which symptom criteria were met. Exacerbation frequency per year was calculated for each patient based on days of observation with daily diary card. Sputum and blood samples were collected prior to commencing any additional systemic therapy according to standard guidelines. Clinical demographics can be seen in Table 1. All exacerbations were considered moderate, and required additional systemic therapy. None of the patients required hospital admission.

### Lower airway bacterial colonisation

Paired sputum samples were available in 77 patients. Sputum was processed as previously described [16] and frozen at − 80 °C. Sputum samples were heat-killed and washed in PBS. DNA was extracted using 10% Chelex 100 and PCR performed to detect the potentially pathogenic micro-organisms (PPMs) *S. pneumoniae*, *H. influenzae* and *M. catarrhalis*. The limit of detection was 10^4^ colony-forming units/ml. The presence of one of more PPMs in sputum was used to define those patients with LABC.

### Macrophage isolation

Monocyte-derived macrophages (MDM) were generated from monocytes isolated from peripheral blood mononuclear cells using a Percoll gradient and adherence technique, followed by culture in 2 ng/ml GM-CSF for 12 days to generate MDM as described previously [2].

### Bacteria

Non-typeable *H. influenzae* (NCTC 1479) and S*. pneumoniae* serotype 10692 were grown as previously described [11]. Bacteria were fluorescently labelled using Alexa-fluor 488 NHS ester and incubated overnight. Labelled bacteria were washed repeatedly in PBS to remove unbound label, resuspended in PBS and stored at − 20 °C.

### Phagocytosis

Bacterial stocks were sonicated then added to macrophages at 5 × 10^8^ CFU/ml, and incubated at 37 °C for 4 h, after which supernatant was stored at − 80 °C for cytokine analysis. Unbound prey was removed by washing in PBS, and extracellular fluorescence quenched with trypan blue (1%v/v) for 1 min. Fluorescence (expressed as relative fluorescence units (RFU) was read by fluorimetry at excitation λ480nm and emission λ520nm. Intracellular phagocytosis has previously been confirmed by confocal microscopy [11].

### Cell viability

Following phagocytosis, cell viability was measured using thiazolyl blue tetrazolium bromide (MTT) assay as previously described [17]. Phagocytosis had no effect on cell viability.

### Cytokine analysis

CXCL-8 and TNFα were measured in MDM supernatants by ELISA according to manufacturer’s instructions (Sigma Aldrich, UK).

### Statistical analysis

Data were analysed using GraphPad PRISM version 6.0 (GraphPad Software Inc., San Diego, CA, USA). Differences between groups were analysed by Mann–Whitney U Test, Wilcoxon-matched pairs, or with a Kruskal–Wallis test with adjustment for multiple post-hoc comparisons, depending on the sample population being investigated. Associations between variables were investigated using linear regression analysis and Pearson’s Correlation.

## Results

### Stability of phagocytosis

**Fig. 1:**
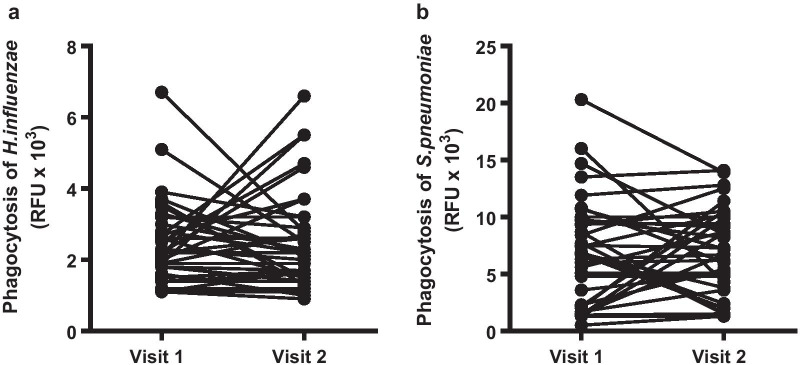
Stability of MDM phagocytosis to **a** *H. influenzae* and **b** *S. pneumoniae* over time. Repeat stable samples were collected over a median time of 9.5 [6.0–14.0] months. Phagocytosis of fluorescently labelled bacteria was measured after 4 h by fluorimetry. N = 36. Data show individual data points, analyzed by Wilcoxon-matched pairs test

To assess the stability of phagocytosis by MDM over time, repeat samples were taken from COPD patients a median of 9.5 (6–14) months apart, and phagocytosis measured. There was no significant change in phagocytic capacity to either *H. influenzae* or *S. pneumoniae* over time (Fig. 1).

### Analysis of clinical factors that influence macrophage function

To determine whether the clinical phenotype of COPD patients affects the function of their MDM, correlations were drawn between patient demographics and phagocytic ability.

**Fig. 2:**
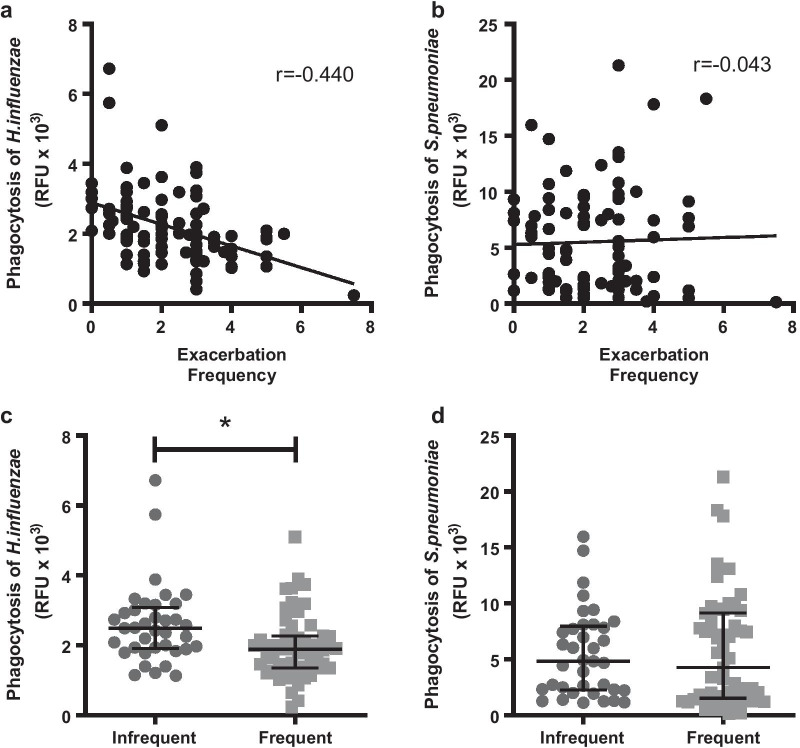
Relationship between MDM phagocytosis and exacerbation frequency. **a** *H. influenzae* **b** *S. pneumoniae* showing exacerbation frequency per year as a continuous variable (n = 92). **c** *H. influenzae* **d** *S. pneumoniae* showing exacerbation status as ‘frequent exacerbator’ ≥ 2 exacerbations/year (n = 55), or infrequent exacerbator with < 2 exacerbations per year (N = 37). Data presented as median and interquartile range. *p < 0.05 Mann Whitney test

*Exacerbation frequency* Phagocytosis of *H. influenzae* was negatively correlated with exacerbation frequency (p < 0.001; r = − 0.440, Fig. 2a), but there were no significant associations between annual exacerbation frequency and phagocytosis of *S. pneumoniae* (p > 0.05, Fig. 2b). Patients were then grouped into frequent and infrequent exacerbators, defined as ≥ 2 exacerbations/year and < 2 exacerbations/year respectively. MDM phagocytosis of *H. influenzae* was significantly lower in the frequent exacerbator (1.9 ± 0.1 RFU × 10^3^) patients than the infrequent exacerbators (2.5 ± 0.2 RFUx10^3^, p < 0.01, Fig. 2c). Exacerbation frequency did not affect phagocytosis of *S. pneumoniae* (Fig. 2d).

**Fig. 3:**
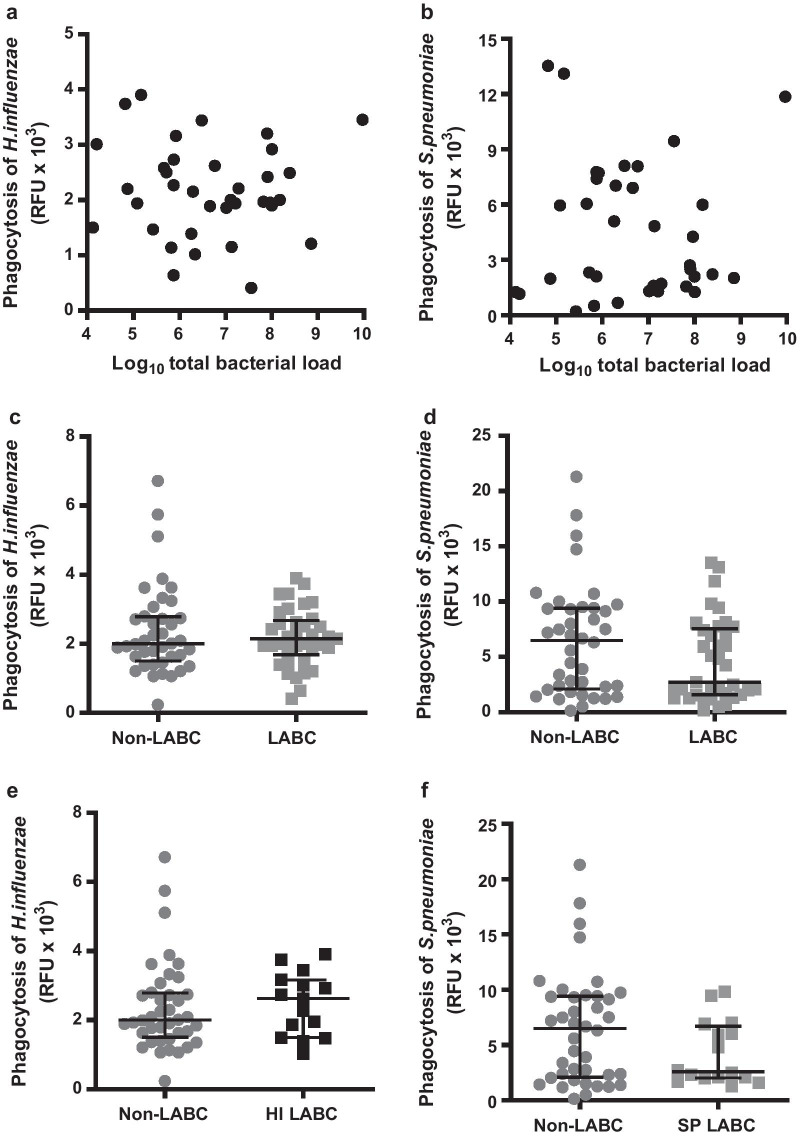
The effect of LABC on MDM phagocytosis. **a** *H. influenzae* **b** *S. pneumoniae* showing Log_10_ bacterial load (n = 37). **c*** H. influenzae* **d** *S. pneumoniae* showing patients with no detectable LABC (n = 40) or with LABC (n = 37). **e** *H. influenzae* showing patients with no detectable LABC (n = 40) or with only *H. influenzae* detectable (n = 15). **f** *S. pneumoniae* showing patients with no detectable LABC (n = 40) or with only *S. pneumoniae* detectable (n = 16). Analysed by Mann Whitney test

*Clinical parameters* There was no significant relationship between the ability of MDM to phagocytose *H. influenzae* or *S. pneumoniae* with age (Additional file 1: Fig. 1A, B), severity of lung disease as measured by FEV_1_% predicted (Additional file 1: Fig. 1C, D) or airway obstruction by FEV_1_/FVC ratio (Additional file 1: Fig. 1E, F); smoking history as measured by pack years (Fig. 3g–h); or by current smoking status (Additional file 1: Fig. 1I, J).

*Treatment* 83.7% (77/92) of patients were using an inhaled corticosteroid (ICS), with a median daily dose of 1000 [1000–2000] μg beclomethasone dipropionate equivalent dose. No significant difference was seen in the phagocytic capacity of either *H. influenzae* (Additional file 2: Fig. 2A) or *S. pneumoniae* between patients using different ICS formulations (Additional file 2: Fig. 2B).

87.0% (80/92) of patients were using a long-acting muscarinic antagonist (LAMA), and in all cases, patients were using 18 μg tiotropium bromide daily. There was no significant effect of concurrent LAMA use on the ability of MDM to phagocytose either bacteria (Additional file 2: Fig. 2C, D). 72/92 patients were using a long-acting β2 agonist (LABA), of which 15 were using formoterol fumerate 24 μg daily and 57 were using salmeterol 100 μg daily. There was no significant effect of concurrent LABA use on the ability of MDMs to phagocytose either bacteria (Additional file 2: Fig. 2E, F).

### Lower airway bacterial colonisation

One or more PPMs were identified by qPCR in 48% (37/77) of stable state patients, and these patients were defined as having evidence of LABC. Mono-microbial isolation with *S. pneumoniae* was identified in 16/37 (43%) of patients, with mono-microbial isolation of *H. influenzae* identified in 15/37 (41%) of patients. Only 1/37 (3%) patients had evidence of mono-microbial *M. catarrhalis* isolation. 5/37 (14%) patients had evidence of polymicrobial detection; three patients had both *H. influenzae* and *S. pneumoniae* detected, one patient had *H. influenzae* and *M. catarrhalis *detected and one patient had all three PPMs identified by qPCR detected.

There was no correlation with macrophage phagocytosis and total bacterial load (Fig. 3a, b). There was also no significant difference in phagocytosis between patients who had evidence of LABC with any bacteria, compared to those patients without evidence of LABC (Fig. 3c, d). In order to determine whether the ability of MDM to phagocytose either *H. influenzae* or *S. pneumoniae* was related to colonisation by the same pathogen, patients without evidence of LABC were compared to those with either mono-microbial *H. influenzae* (n = 15) or *S. pneumoniae* (n = 16). No significant difference was seen between the phagocytic capacity of *H. influenzae* (Fig. 3e) or *S. pneumoniae* (Fig. 3f) and those patients without any evidence of LABC.

### Exacerbation

39 patients were further studied at the onset of exacerbation as well as 2 weeks post-exacerbation, and in the stable state. The stable clinical demographics of these patients are shown in Table 1.

### Effect of exacerbation on macrophage phagocytosis

**Fig. 4:**
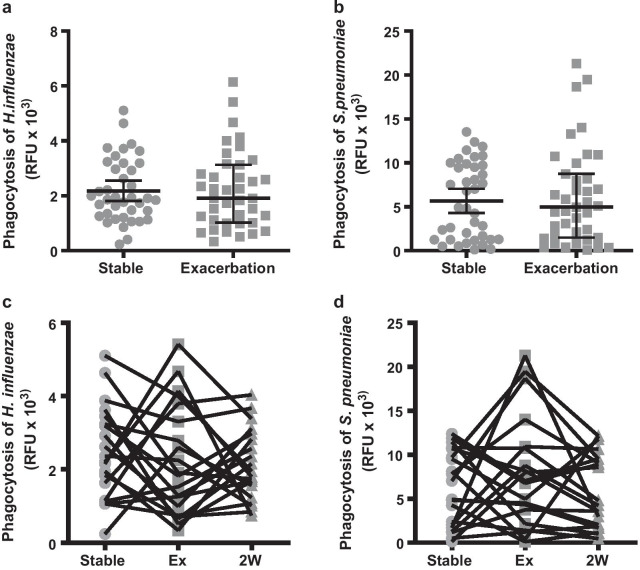
The effect of exacerbation on MDM phagocytosis. **a** *H. influenzae* **b** *S. pneumoniae* showing paired samples taken at stable state or at exacerbation presentation (n = 39). Analysed by Mann Whitney test. Box plots represent the median and interquartile range. **c** *H. influenzae* **d** *S. pneumoniae* showing paired samples taken at stable visit, exacerbation presentation (Ex) or exacerbation follow up at 2 weeks (2 W). Analysed by Friedman test (n = 20)

There was no significant difference in MDM phagocytosis of either bacteria at stable state or at the onset of exacerbation within the same patient (Fig. 4a, b). In order to further understand the effect of exacerbation of MDM function, 20 patients were further sampled 2 weeks after exacerbation presentation. All patients had been given systemic treatment with a 7-day course of both antibiotics and oral corticosteroids at exacerbation presentation. At the 2-week follow-up visit, only 9 (45%) patients had fully recovered from their exacerbation. In the 20 patients with paired stable-exacerbation-2-week samples, no significant difference was observed between the phagocytic capacity of either bacterial species (Fig. 4c, d).

### Effect of exacerbation on MDM cytokine release

To determine whether there was any change in cytokine release between exacerbation status, release of CXCL8 and TNFα by MDM in response to bacterial phagocytosis were compared between the paired stable and exacerbation states in 37 patients.

**Fig. 5:**
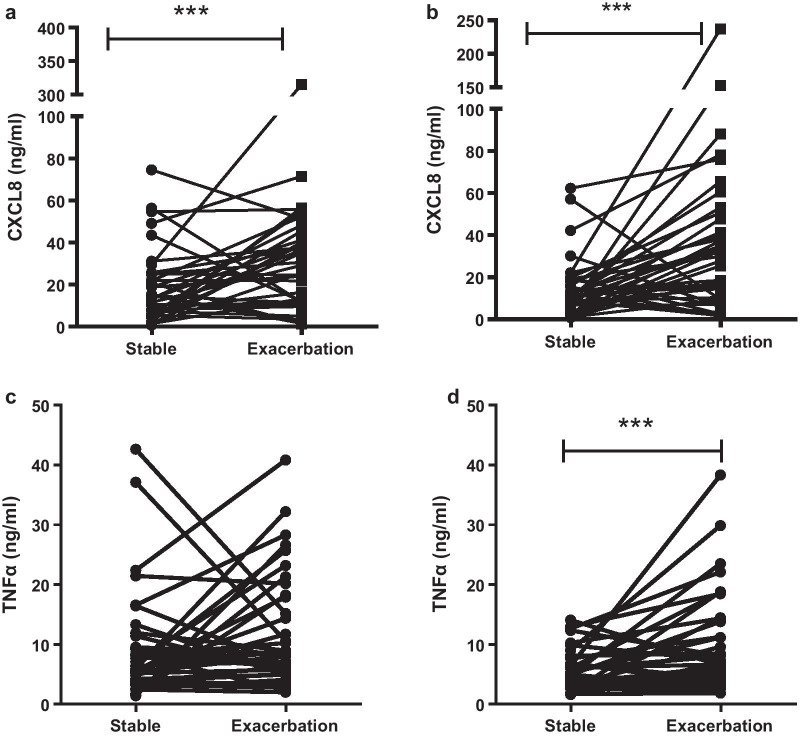
The change in cytokine release between stable and exacerbation states following MDM incubation **a** *H. influenzae* **b** *S. pneumoniae* showing CXCL-8 release. **c** *H. influenzae* **d** *S. pneumoniae* showing TNFα release. Paired samples were taken from 37 patients. ***p < 0.001 analysed by Mann Whitney test

In the stable state, CXCL8 was released from MDM following incubation with either *H. influenzae* or *S. pneumoniae*. In MDM taken at exacerbation, the release of CXCL-8 significantly increased following incubation with either bacteria (p < 0.001, Fig. 5a, b).

In the stable state, TNFα was released from MDM following incubation with either *H. influenzae* or *S. pneumoniae*. In MDM at exacerbation, the release of TNFα significantly increased following incubation with *S. pneumoniae* (p < 0.01, Fig. 5d), but no change was seen in response to *H. influenzae* (Fig. 5c).

## Discussion

Alveolar macrophages are the primary phagocytic and immune-modulating innate immune cell of the airways, whose main function is to phagocytose and destroy invading pathogens. In COPD, alveolar and monocyte-derived macrophage function is altered, resulting in reduced phagocytosis of bacteria [2, 3], apoptotic cells [4] and fungi [5] and an increased release of pro-inflammatory mediators [6]. The mechanism behind this defect is unclear; however, targeting macrophage function may provide a future treatment for patients with COPD. Previous studies on macrophage function have suggested that the defect in phagocytosis may contribute to LABC, lung function decline and exacerbation development, but this has not been fully studied. Our data, using MDM, shows that impaired phagocytosis in COPD macrophages is associated with exacerbation frequency, and that these macrophages are pro-inflammatory, and so they may contribute to disease progression.

We showed that the ability of MDM to phagocytose bacteria was unchanged or stable over time, suggesting that measuring phagocytosis in stable disease is not influenced by disease progression in the short term. This is important when considering data that utilizes ex vivo macrophage function in this disease. The heterogeneity in responses in this data set, while overall non-significant, may reflect the heterogeneity of the COPD population seen in clinical practice.

Phagocytosis of clinically relevant bacteria was not significantly associated with any demographic data. There was no correlation with patient age or with severity of lung disease measured by FEV_1_% predicted, or by FEV_1_/FVC ratio. This confirms previous studies showing no association with alveolar macrophage phagocytosis and FEV_1_% predicted [18]. There was also no associated of phagocytosis with current smoking status, or pack year history. In healthy smokers, smoking status and pack year history has been shown to affect macrophage recognition of bacteria [19, 20] and phagocytosis compared to non-smokers [2, 3, 19]. In our study, only macrophages from COPD patients were studied, and all patients had considerable exposure to tobacco smoke with a median pack year history of 50. This suggests that the presence of disease, rather than the amount of lung function decline, or the amount of smoke exposure is the determining factor of macrophage function and suggest that it may be a fundamental causative mechanism of the disease. The bacteria used in this study were characterized clinical isolates, allowing the comparison of macrophage function to be studied. However, it is clear that there is also variation in bacterial isolates, and the possibility of patients responding to their own isolates, or more virulent strains of bacteria differently. This may contribute to the severity of disease or frequency of exacerbations, and warrants further study.

Studies have shown that corticosteroids may further suppress phagocytosis observed in COPD patients and that this could increase the risk of pneumonia, despite a reduction in exacerbation frequency in those taking ICS [21, 22]. However, a recent study from our laboratory has shown no effect of budesonide or fluticasone propionate on MDM phagocytosis in vitro [23], suggesting that this is not the case. In the current study, all patients were taking inhaled corticosteroids, but we found no relationship between MDM phagocytosis and ICS formulation or dose, confirming our previous in vitro study.

LAMA and LABA are key maintenance therapies in COPD. While they are used primarily for their bronchodilatory effects, both medications have been shown to reduce the underlying chronic inflammation characteristic of COPD [24]. In addition, formoterol but not salmeterol, has been shown to reduce inflammatory cytokine release from MDM from healthy patients [17]. We found no effect on either concurrent LAMA or LABA use on MDM phagocytosis, which confirms previous studies [2, 25] and suggests that the defect in MDM phagocytosis is inherent and not due to pharmacological intervention.

We found a significant relationship between decreasing MDM phagocytosis of *H. influenzae* and worsening exacerbation frequency, although no significant relationship was observed with *S. pneumoniae*. *H. influenzae* is the most common bacterial pathogen isolated in both stable and exacerbated COPD [26, 27]. A key pathogenic mechanism of *H. influenzae* is its ability to persist in the lower airways, adhering to the epithelium or residing between the epithelial and subepithelial tissues, which together with impaired phagocytosis contributes to its persistence in the airways [28, 29]. Therefore, if defective macrophage phagocytosis potentially increases the risk of *H. influenzae* colonization due to insufficient removal, the subsequent higher chronic airway inflammation may contribute to the increase in exacerbation susceptibility observed. This idea is supported by a decrease in MDM efferocytosis of eosinophils by frequent exaberbators, which may lead to eosinophil necrosis and associated tissue damage [30]. *S. pneumoniae* is less commonly seen in COPD exacerbations but its presence in the COPD lung is associated with increased exacerbation frequency [26]. While it is clear that phagocytosis of this pathogen is decreased in COPD macrophages [2, 11], which may contribute to its prevalence in the COPD lung, we found no association of its phagocytosis with worsening exacerbation frequency, suggesting that other mechanisms are also at play, for example neutrophil phagocytosis and NETosis.

Some patients, irrespective of disease severity, are particularly susceptible to exacerbations, and these patients have been termed ‘frequent exacerbators’ [31]. As acute exacerbations of COPD are major determinants of the mortality and morbidity associated with COPD, they are a key clinical outcome in pharmacological studies evaluating new therapies. Thus, potentially correcting the defective macrophage phagocytosis, which appears to be associated with increased exacerbation frequency in patients with COPD, may result in their reduction and improved clinical outcomes.

LABC is associated with the pathogenesis of airway inflammation and exacerbations of COPD [32]. Although macrophage phagocytosis is the most important cellular component to clear particulate matter, including bacteria, it is not the only mechanism involved. It is possible that the attenuated phagocytosis would be more likely to be associated with the presence and greater load of bacteria. However, we found no association between MDM phagocytosis and total bacterial load, or between patients with detectable LABC and those without. Furthermore, we found no significant differences in phagocytosis of the same pathogen that was present in isolation in the sputum. This suggests that macrophage phagocytosis is not the only determining factor of LABC and that a more complex combination of events contributes to its development.

Risks prevent collection of alveolar macrophages by invasive sampling with bronchoscopy during acute exacerbations. The MDM model is well characterized, and maintains the same impaired phagocytosis phenotype as alveolar macrophages [2, 11], allowing macrophage function to be studied during exacerbation due to the ease of blood sampling. We found that there was no change in MDM function between stable and exacerbation state within the same patient, and that recovery of exacerbation of 2 weeks also did not affect MDM function. It is likely therefore, that macrophage phagocytic function remains unchanged during exacerbation but increased bacterial load may drive increased pro-inflammatory mediator release from macrophages and result in increased tissue damage post exacerbation.

To measure this, we measured cytokine release from MDM at stable state and during exacerbations. We found a significant increase in both CXCL8 and TNFα release from MDM following bacterial phagocytosis during exacerbation compared to the stable state. This would result in increased activation of neutrophils in the lungs [33] and contribute to the increase of airway inflammation [34] and oxidative stress [35] seen during exacerbation. Importantly, these results are seen in MDM differentiated from blood monocytes isolated at the onset of exacerbation and cultured for 12 days post sampling. The fact that these cells still show elevated cytokine release shows that there is systemic priming of monocytes during exacerbation. In the lungs, these primed monocytes are recruited and differentiate into hyper-activated macrophages which are more inflammatory then at stable state. This may contribute to worsening lung damage and lung function decline post exacerbation, pro-longing the resolution of inflammation and recovery from exacerbation.

## Conclusion

In conclusion, our study shows that defective COPD macrophage phagocytosis of *H. influenzae* is associated with increased exacerbation frequency, likely a result of an intrinsic defect within the macrophage. This defect appears to confer susceptibility to exacerbations, but cannot be fully explained by the relationship to airway pathogen presence. During exacerbations, while macrophage phagocytosis does not change, the cells are systemically primed to increase pro-inflammatory mediator release. This suggests an intrinsic defect in macrophage function and systemic contribution to exacerbation. Targeting systemic inflammation and macrophage function during exacerbation may limit lung damage in COPD.

## Supplementary Information

**Additional file 1: Figure 1.** Relationship between clinical demographics and MDM phagocytosis. (A) *H. influenzae* (B) *S. pneumoniae* showing age (years) (C) *H. influenzae* (*D*) *S. pneumoniae* showing FEV_1_% predicted. (E) *H. influenzae* (F) *S. pneumoniae* showing FEV_1_/FVC ratio. (G) *H. influenzae* (H) *S. pneumoniae* showing smoking (pack year history). (I) *H. influenzae* (J) *S. pneumoniae* showing current smoking status (ex-smoker n = 60, smoker n = 32). Data analysed by Spearman rank correlation (n = 92).

**Additional file 2: Figure 2.** The effect of different treatments on MDM phagocytosis. (A) *H. influenzae* (B) *S. pneumoniae* showing patients taking no ICS (n = 15), beclomethasone (BM, n = 4), budesonide (BU, n = 14) or fluticasone propionate (FP, n = 59) analysed by Kruskal Wallis test. (C) *H. influenzae (D) S. pneumoniae* showing patients taking long acting muscarinic agonists (LAMA, n = 12 vs. 80). (E) *H. influenzae* (F) *S. pneumoniae* showing patients taking long-acting beta agonists (LABA, n = 20 vs. 72). Analysed by Mann Whitney test.

## Data Availability

The data that support the findings of this study are available from COPDMAP consortium but restrictions apply to the availability of these data, which were used under license for the current study, and so are not publicly available. Data are however available from the authors upon reasonable request and with permission of COPDMAP consortium.

## Abbreviations

LABA: Long activing β2 agonist
LABC: Lower airway bacterial colonisation
LAMA: Long activing muscarinic antagonist
COPD: Chronic obstructive pulmonary disease
FEV1: Forced expiratory volume in one second
FVC: Forced vital capacity
MDM: Monocyte-derived macrophage
PPMs: Potentially pathogenic microorganisms
RFU: Relative fluorescence units
