# Supplementary figures and images for: Defective monocyte-derived macrophage phagocytosis is associated with exacerbation frequency in COPD
Source: Respir Res. 2021 Apr 20;22:113. doi: 10.1186/s12931-021-01718-8 (PMC8059282; doi:10.1186/s12931-021-01718-8)

## Slide 1
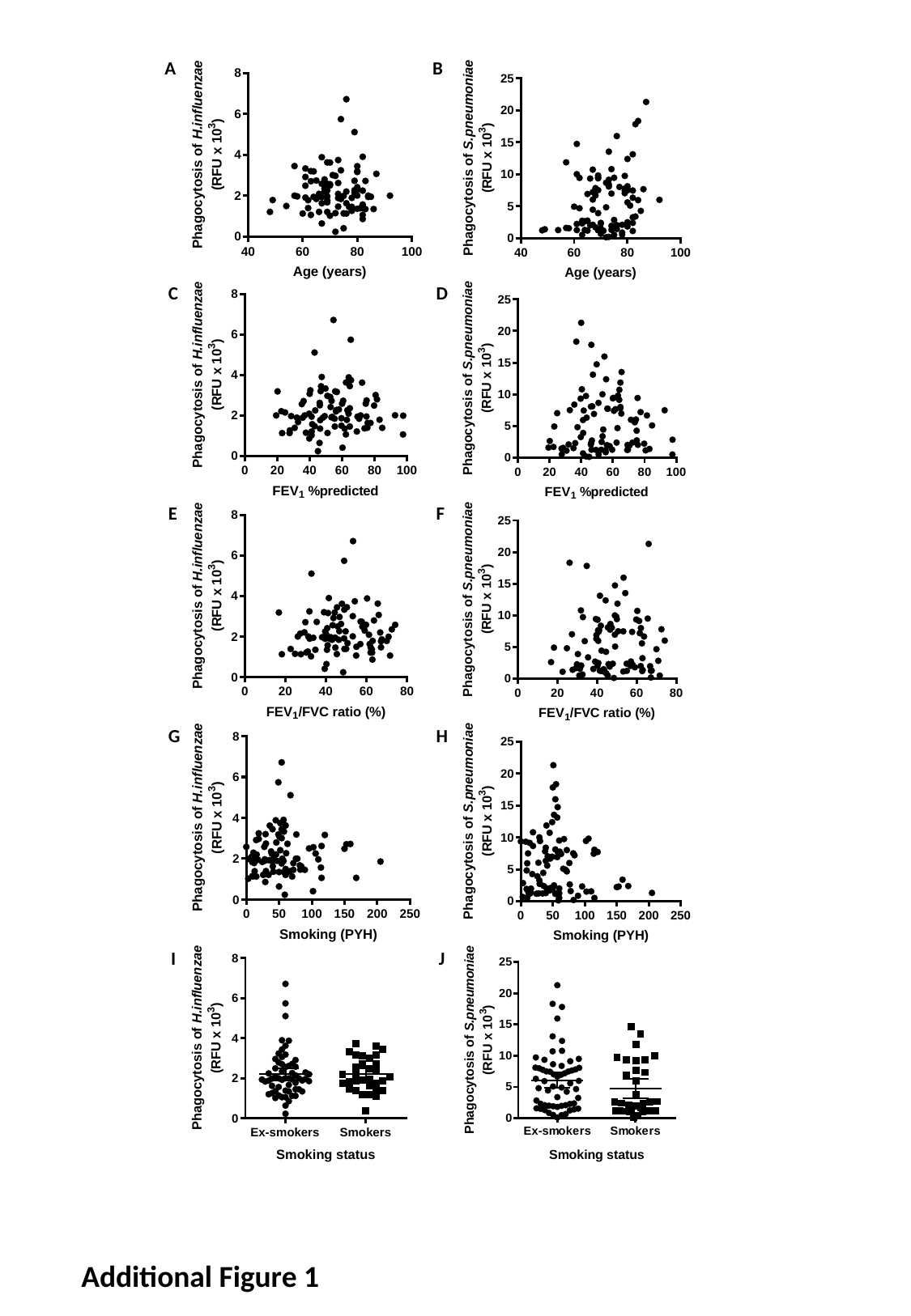

A
B
C
D
E
F
G
H
I
J
Additional Figure 1

Supplement: Supplementary file 1 — Additional file 1: Figure 1. Relationship between clinical demographics and MDM phagocytosis. (A) H. influenzae (B) S. pneumoniae showing age (years) (C) H. influenzae (D) S. pneumoniae showing FEV1% predicted. (E) H. influenzae (F) S. pneumoniae showing FEV1/FVC ratio. (G) H. influenzae (H) S. pneumoniae showing smoking (pack year history). (I) H. influenzae (J) S. pneumoniae showing current smoking status (ex-smoker n = 60, smoker n = 32). Data analysed by Spearman rank correlation (n = 92). [file 12931_2021_1718_MOESM1_ESM.pptx]

## Slide 1
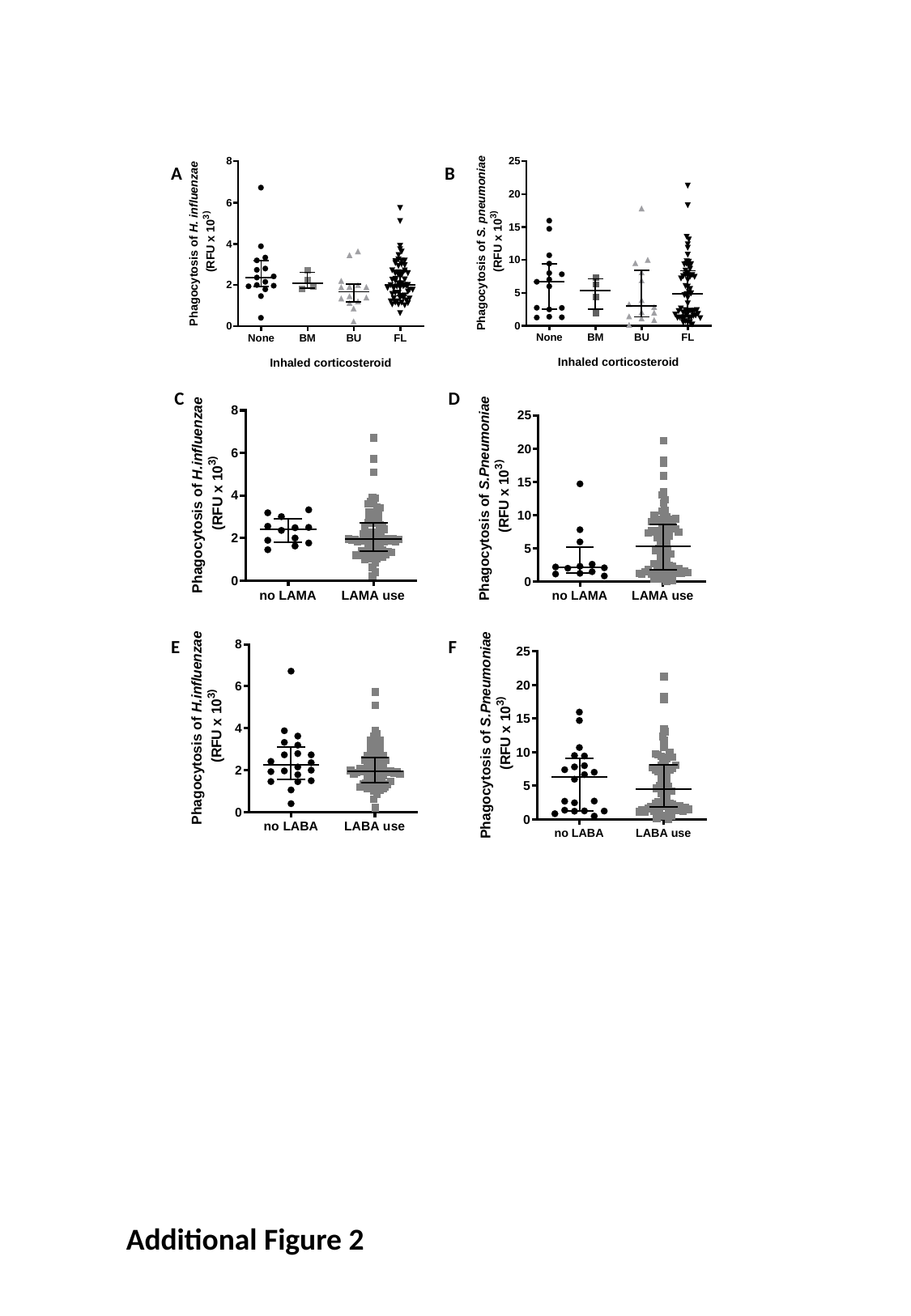

A
B
C
D
E
F
Additional Figure 2

Supplement: Supplementary file 2 — Additional file 2: Figure 2. The effect of different treatments on MDM phagocytosis. (A) H. influenzae (B) S. pneumoniae showing patients taking no ICS (n = 15), beclomethasone (BM, n = 4), budesonide (BU, n = 14) or fluticasone propionate (FP, n = 59) analysed by Kruskal Wallis test. (C) H. influenzae (D) S. pneumoniae showing patients taking long acting muscarinic agonists (LAMA, n = 12 vs. 80). (E) H. influenzae (F) S. pneumoniae showing patients taking long-acting beta agonists (LABA, n = 20 vs. 72). Analysed by Mann Whitney test. [file 12931_2021_1718_MOESM2_ESM.pptx]
